# Evaluation of the trauma criteria from the Norwegian national trauma plan: an observational study from a regional trauma centre

**DOI:** 10.1186/s13049-026-01634-0

**Published:** 2026-05-27

**Authors:** Guro Bjørke Buljo, Kenneth Thorsen, Thomas Geisner

**Affiliations:** 1 Department of Surgery, Voss Hospital, Helse Bergen, Voss, Norway; 2 Department of Clinical Medicine, University of Bergen, Bergen, Norway; 3 Section for Traumatology, Surgical Clinic, Stavanger University Hospital, Stavanger, Norway; 4 Department of Gastrointestinal Surgery, Stavanger University Hospital, Stavanger, Norway; 5 Department of Gastrointestinal Surgery, Haukeland University Hospital, Bergen, Norway

**Keywords:** Trauma, Trauma team, Trauma team activation protocol, Overtriage, Undertriage, Mortality, Injury severity

## Abstract

**Background:**

The national trauma plan defines common Norwegian criteria dictating which patients are met by a trauma team. Precise criteria are important to avoid undertriage and overtriage. Previous studies have indicated that both overtriage and undertriage fail to meet the goals of the Norwegian Trauma Plan, and the aim of the study was to investigate the performance of the new national criteria.

**Methods:**

This is a retrospective cohort study of patients included in the trauma registry at Haukeland University Hospital between 2017 and 2021. Injury Severity Score (ISS) was extracted from the registry. Prehospital records were manually reviewed to determine which trauma criteria were met. The aim was to analyse how well the national trauma criteria predicted ISS > 15, and if national goals of overtriage and undertriage were met.

**Results:**

A total of 2508 patients were eligible for analysis, whereas 384 had an ISS > 15, with a total overtriage of 81.1%. Forty-four patients had an ISS > 15 without meeting the criteria, resulting in an undertriage of 11.5%. If hypothetically *4 Patient factors* was analysed as an individual criteria group, undertriage would have been 2.9%. Patients who did not meet any criteria had an overtriage of 97.6%. *Mechanism of injury (MOI)* predicted ISS > 15 in nine of 333 patients, resulting in an overtriage of 97.3% for this criteria group. Hypothetical removal of the MOI criteria except *3.6 Fall* would in this data set eliminate 255 Trauma team activations (TTA) among patients with ISS < 15.

**Conclusions:**

Overtriage was high overall and across all criteria groups. Patients who triggered a TTA despite not meeting any criteria also demonstrated substantial overtriage. To optimize resource use, criteria with low positive predictive value could be assigned fewer resources or revised to reduce unnecessary activations. Our findings support re-evaluation of specific criteria, particularly those related to mechanism of injury.

## Background

The Norwegian trauma plan provides guidelines for the Norwegian trauma care, hereunder national trauma criteria and quality predictors [1]. Before national Norwegian trauma criteria were defined in 2017, different regions in Norway used different criteria [2]. The national criteria were based on Centers for Disease Control and Prevention (CDC) Field triage and an expert panel [1, 2]. The aim is to prehospitally identify which patients have a severe injury, consequently needing trauma team activation (TTA) [3, 4]. The trauma team includes, at minimum, surgical team leader, examining surgeon, anesthesiologist, emergency department nurse, nurse anesthetist, scrub nurse and radiographer, in accordance with national guidelines[1]. The criteria are divided into four different criteria groups with descending priority, *1 Physiology*, *2 Anatomical injury*, *3 Mechanism of injury* and *4 Patient factors* [1, 3, 4].

The trauma criteria hence constitute a tool to identify severely injured patients in need of a trauma team activation (TTA), while simultaneously excluding patients with minor injuries, to reduce unnecessary TTA[3, 4]. Several methods are used to define severe injury, the common factor being their retrospective nature [3–5]. The most frequently used is Abbreviated Injury Scale (AIS), defining 9 body areas where the sum of the squares of the three highest scores gives an Injury Severity Score (ISS) of 0–75 [5]. A major study found that ISS > 15 was associated with a 10% mortality, thereby defining ISS > 15 as severe injury and need for TTA to reduce morbidity and mortality [4].

Overtriage is defined as patients with an ISS < 15 that are met by a trauma team, whilst undertriage is defined as patients with an ISS > 15 who do not get a TTA [3, 4, 6]. The Norwegian trauma protocol defines quality predictors that set a goal of overtriage less than 50% and undertriage less than 5% [1]. Triage performance must balance overtriage and undertriage, to ensure both efficient use of resources and patient safety [3]. Several Norwegian studies have found overtriage and undertriage to be out of line with these goals [7–15] yet these studies have reviewed the regional criteria in use before 2017.

Both previous research and the consensus among trauma team personnel was that there was a high rate of overtriage contributing to inefficient use of resources, and that the trauma criteria contributed to this overtriage. We therefore aimed to investigate the predictive ability of the trauma criteria.

## Materials and methods

### Aim

The aim of this study was to investigate the performance of the trauma criteria, the criteria groups and the single criteria in patients admitted to the regional trauma centre in Western Norway, and to evaluate if the national trauma plan should be reevaluated.

### Design

This is a retrospective cohort study of all patients included in the trauma registry at Haukeland University Hospital (HUH) 2017–2021.

### Ethics

An application for ethical approval was submitted to the Regional Committee for Medical and Health Research Ethics (REK); however, the study was classified as a quality assurance project. The study was accepted by the local data protection officer.

### Clinical setting

Haukeland University Hospital (HUH) is a regional trauma centre in Norway. It has a catchment area of approximately 500,000 residents, and approximately 600 annual TTAs. The trauma registrars are certified by the Association for the Advancement of Automotive Medicine Abbreviated Injury Scale (AIS) course.

### Material

The local trauma registry includes all TTA patients irrespective of ISS, as well as injured patients that did not trigger a TTA but had an ISS > 9, a penetrating injury or an isolated head injury with Abbreviated Injury Scale (AIS) ≥ 3, as described in national registry inclusion criteria [1]. All the patients included in the trauma registry in the period were included in the study. ISS was extracted from the registry. Prehospital records were manually reviewed to register which trauma criteria were met. Transferred patients were excluded because in-hospital evaluation deemed them unsuitable for validation of prehospital criteria.

### Definitions

Severe injury was defined as an injury severity score (ISS) > 15. Overtriage was defined as TTA based on a criterion when having an ISS < 15. Undertriage was defined as ISS > 15 with the lack of a criterion launching TTA.

### Statistics

Statistics are presented as counts and percentages (%). Estimates of positive predictive value (PPV), sensitivity, overtriage, and undertriage were calculated using the Cribari method. All estimates are reported with corresponding 95% Wilson confidence intervals (CI). Statistical analyses were performed using IBM SPSS Statistics. For all analyses, only the highest-priority criterion per patient was registered, ensuring that the contribution of each criterion could be assessed independently. Including all criteria would obscure the analysis of which criteria were redundant.

## Results

**Fig. 1:**
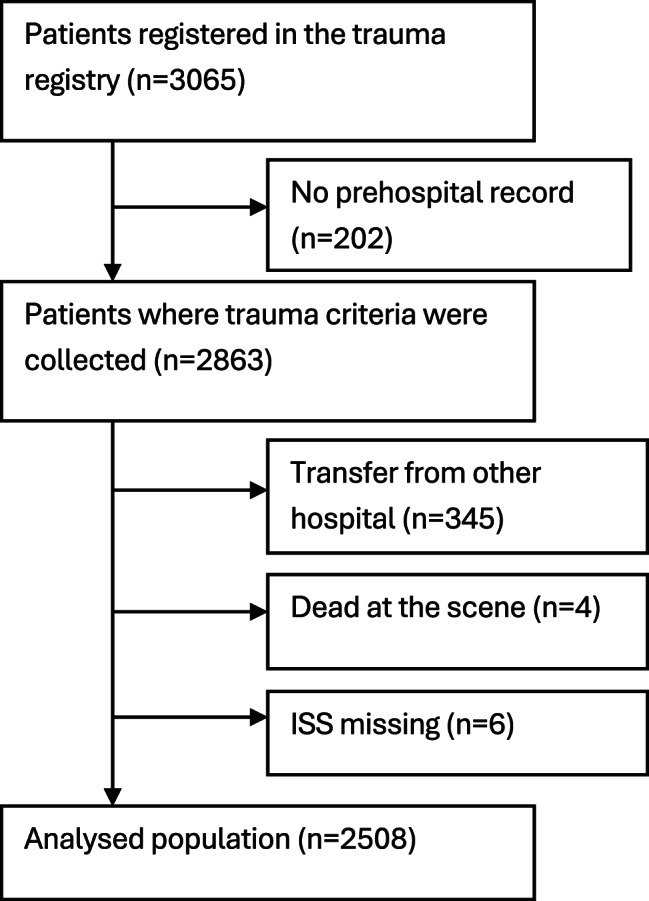
Overview of the included patients

Following exclusion, 2508 patients were included in the analyses (Fig. 1). A total of 1802 patients met the criteria when excluding criteria group *4 Patient factors*, that are not considered criteria that can individually trigger TTA. This gave a total overtriage of 81.1%. There were 384 patients with ISS > 15 (15.3%), of which 11 did not meet any criteria and 33 met criteria only in *4 Patient factors*. This gave a total undertriage of 11.5%. Hypothetically, if criteria group *4 Patient factors* could individually trigger TTA, undertriage would have been 2.9% (Table 1).

**Table 1:** Sensitivity, undertriage, PPV and overtriage for the whole protocol

	Counts	Est. (%)	95% CI
**Current protocol**			
Sensitivity	340/384	88.5	85.0-91.4
Undertriage	44/384	11.5	8.6–15.0
PPV	340/1802	18.9	17.1–20.7
Overtriage	1462/1802	81.1	79.3–82.9
**Hypothetical protocol** **(With criteria group 4)**			
Sensitivity	373/384	97.1	94.9–98.4
Undertriage	11/384	2.9	1.6–5.1
PPV	373/2058	18.1	16.5–19.8
Overtriage	1685/2058	81.9	80.2–83.5

Sensitivity = P(met one or more criteria | ISS > 15)

Undertriage = 1 – sensitivity

PPV = P(ISS > 15 | met one or more criteria)

Overtriage = 1- PPV

In criteria group *1 Physiology*, 664 patients met a criterion, whereby 206 with ISS > 15, which gave an overtriage of 69.0%. In criteria group *2 Anatomical injury*, 805 patients met a criterion, whereby 125 patients with ISS > 15, giving an overtriage of 84.5%. In criteria group *3 Mechanism of injury*, 333 patients met a criterion, whereby 9 with ISS > 15, giving an overtriage of 97.3%. In criteria group *4 Patient factors*, 256 patients met a criterion, whereby 33 with ISS > 15, giving an overtriage of 87.1%. There were 450 patients who did not meet any criteria (18.0%) but still had a TTA. Eleven of these patients had an ISS > 15, giving an overtriage of 97.6% (Table 2).

**Table 2:** Overtriage in different criteria groups

CRITERIA GROUP	TOTAL	ISS < 15	ISS > 15	OVERTRIAGE %	95% CI
1 Physiology	664	458	206	69.0%	65.4–72.4
2 Anatomical injury	805	680	125	84.5%	81.8–86.8
3 Mechanism of injury	333	324	9	97.3%	94.9–98.6
4 Patient factors	256	223	33	87.1%	82.4–90.7
5 Did not meet any criteria	450	439	11	97.6%	95.7–98.6

Within criteria group *1 Physiology*, criteria *1.1 RR < 10 or > 29/min or need for ventilatory support* had the highest PPV at 43.4%. All the criteria within this group had a PPV above 20%, except criteria *1.3 Heart rate > 130* with a PPV of 18%. Criteria *1.6 Hypothermia without normal circulation* only triggered one TTA. Within *2 Anatomical injury* there were several criteria that triggered less than 10 TTAs. *Criteria 2.7 Two or more fractures in long bones* and *2.9 Suspicion of spinal injury* had a PPV above 20% (36% and 38% respectively). *2.4 Thoracic injury* and *2.10 Injury in two or more body areas* triggered the most TTAs, with 227 (PPV 19%) and 327 (PPV 10%) respectively. Within *3 Mechanism of injury* criteria 3.1 to 3.4 involves a vehicle crash, these criteria triggered TTA for 146 patients, whereas one had ISS > 15, giving criteria 3.1 a PPV of 1%, whilst the rest of the criteria had a PPV of 0%. Criteria *3.6 Fall from height > 5 m (3 m for children)*, had a PPV of 9%. Seven of the nine patients with ISS > 15 met criterion *3.6 Fall > 5 m*, one met criterion *3.5 Auto vs. pedestrian/bicyclist* and one met criterion *3.1 Vehicle crash > 50 km/h without use of seat belt or triggered airbag*. In criteria group *4 Patient factors* criteria *4.1 Age > 60 years* triggered the most TTAs with 146 patients whereby 24 with ISS > 15 giving a PPV of 16%. In total, ten of the single criteria were the highest prioritised criterion less than ten times, and it is therefore hard to validate their predictive abilities (Table 3).

**Table 3:** Performance of single criteria

Criterion	Total (*n*)	ISS > 15 (*n*)	PPV (%)	95% CI
**1 Physiology**				
1.1 RR < 10 or > 29/min or need for ventilatory support	256	111	43.4	37.3–49.4
1.2 Oxygen saturation (SpO2) < 90% without O2	50	15	30.0	17.3–42.7
1.3 Heart rate > 130/min	44	8	18.2	6.8–29.6
1.4 Systolic BP < 90	54	16	29.6	17.5–41.8
1.5 GCS < 14	259	55	21.2	16.3–26.2
1.6 Severe hypothermia without normal circulation	1	1	100	-
**2 Anatomical injury**				
2.1 Facial trauma with threatened airway	6	2	33.3	0-71.1
2.2 Cranial fracture	8	4	50.0	15.4–84.6
2.3 Penetrating injury to head, neck, torso and extremities proximal to elbow/knee	58	4	6.9	3.8–13.4
2.4 Severe pain in thorax (suspicion of rib fractures)	227	43	18.9	13.8–24.1
2.5 Massive haemorrhage	9	0	0	-
2.6 Crush injury	1	0	0	-
2.7 Two or more fractures in long bones	14	5	35.7	10.6–60.8
2.8 Severe pain in pelvic region (suspicion of pelvic fracture)	85	13	15.3	7.6–22.9
2.9 Suspicion of spinal cord injury	45	17	37.8	23.6–51.9
2.10 Injury in two body sections (head/neck/thorax/abdomen/pelvis/spinal cord/femur)	327	34	10.4	7.1–13.7
2.11 Burn injury, second or third degree > 15% body surface (children > 10%) or inhalation injury	25	3	12.0	0-24.7
**3 Mechanism of injury**				
3.1 Vehicle crash > 50 km/h without use of seat belt or triggered airbag	68	1	1.5	0-4.3
3.2 Vehicle crash where car has rolled over	68	0	0	-
3.3 Vehicle crash, person trapped	9	0	0	-
3.4 Vehicle crash, ejection from automobile	1	0	0	-
3.5 Auto vs. pedestrian/bicyclist	111	1	0.9	0-2.7
3.6 Fall from height >5 m for adults and >3 m for children	76	7	9.2	2.7–15.7
**4 Patient factors**				
4.1 Age > 60 years	146	24	16.4	10.4–22.5
4.2 Age < 5 years	26	1	3.8	0-11.2
4.3 Severe disease (kidney/heart/lung)	3	0	0	-
4.4 Pregnant patient > week 20	5	0	0	-
4.5 Anticoagulants	5	2	40.0	0–83.0
4.6 Under the influence of drugs/alcohol	71	6	8.5	2.0-14.9

The Norwegian protocol has three criteria in *1 Physiology* not found in trauma criteria protocols in other countries, being *1.2 SpO2 < 90%*, *1.3 Heart rate > 130* and *1.6 Hypothermia without normal circulation*. Criteria *1.2 SpO2 < 90%* triggered TTA for 15 patients with ISS > 15, whereby two who did not meet any criteria in *1 Physiology* or *2 Anatomical injury. 1.3 Heart rate > 130* triggered TTA for 8 patients with ISS > 15, whereby one who otherwise did not meet any criteria in criteria group 2 or 3. Criteria *1.6 Hypothermia without normal circulation* triggered TTA for one patient with ISS > 15, but this patient also met criteria in criteria group 2 (Table 3).

A total of 640 (25%) patients were > 60 years, whereby 146 patients with ISS > 15, meaning that 23% of patients > 60 years had a severe injury, versus 12% of patients aged between 5 and 59 years. There were 30 patients > 60 years who died within 30 days (4.6%), and 30 patients aged 5–59 (1.5%) who died within 30-days. Ten of the patients who died had an ISS < 15, which was not seen for the patients 5–59 years, whom all had an ISS > 15. A total of 79 patients were children aged less than 5 years (3.1%), whereby seven had an ISS > 15 (8.9%). Two children died within 30 days, both with ISS > 15. Out of the 11 undertriaged patients, 9 had an isolated head injury.

## Discussion

In this study we demonstrate a very high overtriage at 81.1%. The observed overtriage in all criteria groups exceeded the target of less than 50% defined in the Norwegian trauma plan and is consistent with findings from similar studies [7–16]. Overtriage was predominantly caused by criteria group *3 Mechanism of injury*, individual criteria demonstrating low positive predictive value (PPV), and patients who did not fulfill any criteria. In agreement with previous studies these components of the protocol could be considered for removal or revision. Undertriage (11.5%) was primarily observed in older patients and in patients with isolated head injury.

Previous studies have investigated earlier Norwegian trauma triage protocols [8–16], but to our knowledge only one study has examined the national trauma criteria introduced in 2017 [7]. We therefore consider this study important for evaluating the predictive ability of the criteria and contributing to ongoing efforts to improve them. Although the study is limited by its single-centre design, we believe the findings may be partly generalisable to other Norwegian trauma centres and hospitals with trauma care capabilities, and as such that these data may contribute to the current ongoing revision of the National Trauma Plan.

Criteria *3 Mechanism of injury* had an overtriage comparable to patients who did not meet any criteria at all, with an overtriage of 97.3 and 97.6% respectively. Only one patient with ISS > 15 triggered TTA by meeting a criterion involving a vehicle crash, and the total overtriage for these criteria was at 99.3%. Although high energy trauma may cause occult injuries [3, 17], this was not reflected in our dataset. Several improvements that could influence this have occurred during this period of time. A continuous renewal of the car fleet, improving and updating roads and strict traffic regulations may all contribute to this pattern [18].

The CDC use a threshold of PPV 20% when including criteria in the criteria group *3 Mechanism of injury* [3]. None of the criteria involving a vehicle crash approached this threshold in our study (highest PPV at 0.2%), in line with other Nordic reports [7–16, 19].

Hypothetical exclusion of *3 Mechanism of injury* in our dataset would avoid 324 TTAs for patients with an ISS < 15 at the cost of a 2.1% point increase in undertriage. Retaining only criteria *3.6 Falls > 5 m (> 3 m children)* would have reduced TTAs by 248, with a marginal 0.5% point increase in undertriage. Overtriage would be reduced from 81.1% to 77.4%, representing a very modest reduction, but an annual decrease of 65 TTAs would represent a discernible decreased workload. However, this analysis represents only a hypothetical assessment of protocol modification, and further studies are needed to validate this hypothesis. Of the 8 patients with ISS > 15 who only met a criterion in *3 Mechanism of injury* at the scene, three deteriorated clinically and would likely have triggered physiological or anatomical criteria on reassessment, supporting limited independent value of mechanism criteria.

One fifth of the patients did not meet any criteria, and had an overtriage rate of 97.6%, corresponding to 88 avoidable TTAs annually. The TTAs were activated based on Emergency Medical Services (EMS) personnel’s clinical judgement of the patient, or assessment of the mechanism of injury, sometimes in consultation with the on-call surgeon. Some of these cases may also reflect incomplete prehospital records that did not capture possible fulfilled criteria. This suggests that better compliance with the criteria could reduce overtriage.

Some criteria were applied fewer than ten times over five years, precluding meaningful evaluation. Limited use may reflect overlap with criteria of higher priority. For example, when patients met the criteria *2.5 Massive haemorrhage*, if they had an ISS > 15, they also often met a physiologic criterion such as *1.4 Systolic BP < 90*.

Two physiological criteria were unique to the Norwegian protocol, being *1.3 Heart rate > 130* and *1.6 Hypothermia without normal circulation*. Heart rate (HR) was excluded from Swedish protocols due to limited specificity prehospitally [20]. In our dataset HR was the physiological criteria with the lowest PPV. In 2021 the CDC incorporated shock index (SI) into its triage protocols [21], and the Norwegian protocol has since done the same, excluding HR as a criterion [1].

Patients who met one or more criteria but did not trigger TTA were not classified as undertriage, as the objective was to assess criteria performance rather than compliance. Overall undertriage (11.5%) was lower than yearly reported undertriage at HUS, at 14.6% in 2019 (unpublished) and other studies reporting up to 30%, suggesting that more precise use of the criteria could reduce undertriage [9, 11, 15, 22]. Undertriage could decrease to 2.9% if *4 Patient factors* independently triggered TTA in our dataset, however, this analysis represents only a hypothetical assessment of protocol modification.

Criteria group *4 Patient factors* is not intended to trigger TTA alone, and consequently some uncertainty regarding its practical use remains. This may overestimate its PPV, as not all patients are transported to hospital and therefore included in the registry. This was illustrated when changing from a two-tiered trauma team at Stavanger University hospital, where overtriage increased when TTA was triggered by patients solely meeting criteria in *4 Patient factors* [14].

Undertriaged patients were predominantly patients > 60 years that met only criteria within *4 Patient factors* and isolated head injury presenting with GCS 14–15. Older patients frequently sustain severe injury following low energy trauma, and their physiological response differs from that of younger patients [3]. Given that the physiological criteria rely on substantial deviations from normal physiology, they may be less sensitive in detecting early warning signs in older patients. When revised in 2021 the CDC criteria also included Systolic blood pressure (SBP) < 110 for patients aged ≥ 65 years since older patients could be hypoperfused at lower SBP [21]. Older patients also more frequently use medications like betablockers that can mask changes in physiology [3]. Consistent with prior findings, our data indicate that older patients seem to be more likely to have an occult injury than patients meeting criteria in *3 Mechanism of injury*, the trauma criteria are less sensitive for older patients, and older patients have a higher mortality rate [3, 17, 23].

Undertriage should not merely focus on ISS > 15, since this is quantified by registrars several weeks after the traumatic event. Hence patients in need of emergency treatment are paramount to identify as rapidly as possible, and the ISS is not designed for this nor useful in this context. Patients > 60 years is well recognised as difficult to identify with the trauma criteria [9, 14, 15]. Previous studies have identified a high rate of undertriaged patients to be isolated head injuries after low energy falls in the elderly, but many of these patients are unsuitable candidates for intervention [14, 23]. Hence it is worth questioning if these patients could benefit from another pathway than a TTA and a geriatric trauma team on demand has been implemented in a similar region [23].

Overtriage is traditionally regarded as a resource problem, but allocating scarce resources in smaller hospitals may have adverse effects for other patients. One study demonstrated increased 30-day cardiovascular events among patients admitted with acute coronary syndrome concurrently with TTA [24]. With an overtriage rate of 81.1%, potential consequences for the trauma patients include unnecessary radiation exposure, as the 2023 annual report showed that 81.7% of patients who received a trauma team activation underwent CT imaging [25]. Furthermore, the annual reports from the National Trauma Registry include patient-reported outcomes. In 2024, 13.8% of patients with NISS 0 had not returned to work 12 months after the incident, with similar proportions observed among patients NISS 1–14. The report also indicate elevated rates of depression and anxiety in these groups, suggesting that the traumatic event and subsequent treatment may themselves contribute to long-term patient consequences [25]. Although overtriage is sometimes justified as necessary for maintaining team competence, training should not rely on imprecise screening at the expense of patient safety. Simulation-based training provides an established alternative [26], and it may be argued that an overtriage rate of 50% could be considered adequate for training.

While the Norwegian trauma criteria demonstrate high sensitivity, their specificity remains low. A potential strategy to minimize the resource utilisation associated with overtriage, is giving the criteria groups who most contribute to overtriage less resources. In Sweden a two-tiered trauma team is implemented, like some hospitals in Norway prior to the introduction of national criteria [14, 19]. This is in line with recommendations from the CDC, which state that patients meeting criteria in *3 Mechanism of injury* but not *1 Physiology nor 2 Anatomical injury* are haemodynamically stable, and if occult injury later is identified there is sufficient time to escalate the level of care [3]. Another strategy to mitigate the issue of overtriage is to reduce the problem of overtriage itself by changing the current criteria or utilise different methods of triage. The trauma criteria are binary, with predefined threshold values determining whether abnormal physiological findings meet the criteria. In contrast, several other intrahospital and prehospital triage methods incorporate graded assessments of physiological parameters, allowing for a more nuanced evaluation of physiological changes, for example New Early Warning Score (NEWS). The advantage of binary criteria is that it is rapid and requires no calculations, but more comprehensive methods might be more sensitive for patients with altered baseline physiology, like older patients [3]. The increasingly advanced prehospital tools available today, with the possibility of automated calculations, could enable the use of more advanced triage tools in the prehospital setting.

Following this study, HUH implemented a two-tiered trauma team activation protocol assigning a smaller trauma team to patients meeting only criteria in *3 Mechanism of injury* and *4 Patient factors*, with the aim of reducing undertriage from older patients, and better allocate resources to where they are best needed.

### Limitations and strengths

A strength of the study was that manual review of the prehospital records enabled exclusion of incorrectly triggered mechanism criteria and likely reduction of misclassification. However, the retrospective design introduces risk of bias. Incomplete prehospital records may have led to underestimation of higher priority criteria, leading to an underestimation of overtriage. The decision to activate the trauma team was not consistently made by the documenting clinician, potentially affecting contextual interpretation and leading to discrepancies between documented and actual fulfilled criteria. The trauma registry demonstrates high completeness in capturing patients with trauma team activation (TTA) and makes thorough efforts to include undertriaged patients. However, excluded patients without prehospital records had a higher mean ISS (12.1 vs. 7.5 among included patients), indicating potential selection bias. This is a single-centre design which could limit the generalisability to other trauma systems. Another limitation is that trauma severity was measured solely by ISS. Patients with an ISS < 15 who require emergency interventions will still benefit from a TTA, and this is not reflected in our dataset. Although overtriage and undertriage are commonly evaluated using this approach, it will result in misclassification of certain patients.

## Conclusion

Overtriage was high overall and across all criteria groups. Patients who triggered a TTA despite not meeting any criteria also demonstrated substantial overtriage. To optimize resource use, criteria with low positive predictive value could be assigned fewer resources or revised to reduce unnecessary activations. Our findings support re-evaluation of specific criteria, particularly those related to mechanism of injury.

## Data Availability

All data generated or analysed during this study are included in this published article.

## Abbreviations

AIS: Abbreviated Injury Scale
CDC: The Centers for Disease Control and Prevention
CT: Computed Tomography
EMS: Emergency Medical Services
HR: Heart Rate
SBP: Systolic blood pressure
HUS: Haukeland University Hospital
ISS: Injury Severity Score
MOI: Mechanism of Injury
NEWS: New Early Warning Score
PPV: Positive Predictive Value
REK: Regional Ethics Committee
SI: Shock Index
TTA: Trauma Team Activation
